# Comparison of the first three waves of avian influenza A(H7N9) virus circulation in the mainland of the People’s Republic of China

**DOI:** 10.1186/s12879-016-2049-2

**Published:** 2016-12-05

**Authors:** Nijuan Xiang, A. Danielle Iuliano, Yanping Zhang, Ruiqi Ren, Xingyi Geng, Bili Ye, Wenxiao Tu, Ch ao Li, Yong Lv, Ming Yang, Jian Zhao, Yali Wang, Fuqiang Yang, Lei Zhou, Bo Liu, Yuelong Shu, Daxin Ni, Zijian Feng, Qun Li

**Affiliations:** 1 Chinese Center for Disease Control and Prevention, No. 155 Changbai Road, Changping District, Beijing, China; 2 US Centers for Disease Control and Prevention, Atlanta, GA USA; 3 Jinan Prefecture Center for Disease Control and Prevention, Shandong, China; 4 Shenzhen Prefecture Center for Disease Control and Prevention, Guangdong, China; 5 Tianjin Municipal Center for Disease Control and Prevention, Tianjin, China; 6 Luan Prefecture Center for Disease Control and Prevention, Anhui, China; 7 Xuancheng Prefecture Center for Disease Control and Prevention, Anhui, China; 8 Jiangxi Provincial Center for Disease Control and Prevention, Jiangxi, China; 9 Institute for Viral Disease Control and Prevention, Chinese Center for Disease Control and Prevention, Beijing, China

**Keywords:** Avian influenza, H7N9, China, Pandemic

## Abstract

**Background:**

H7N9 human cases were first detected in mainland China in March 2013. Circulation of this virus has continued each year shifting to typical winter months. We compared the clinical and epidemiologic characteristics for the first three waves of virus circulation.

**Methods:**

The first wave was defined as reported cases with onset dates between March 31-September 30, 2013, the second wave was defined as October 1, 2013-September 30, 2014 and the third wave was defined as October 1, 2014-September 30, 2015. We used simple descriptive statistics to compare characteristics of the three distinct waves of virus circulation.

**Results:**

In mainland China, 134 cases, 306 cases and 219 cases were detected and reported in first three waves, respectively. The median age of cases was statistically significantly older in the first wave (61 years vs. 56 years, 56 years, *p* < 0.001) compared to the following two waves. Most reported cases were among men in all three waves. There was no statistically significant difference between case fatality proportions (33, 42 and 45%, respectively, *p* = 0.08). There were no significant statistical differences for time from illness onset to first seeking healthcare, hospitalization, lab confirmation, initiation antiviral treatment and death between the three waves. A similar percentage of cases in all waves reported exposure to poultry or live poultry markets (87%, 88%, 90%, respectively). There was no statistically significant difference in the occurrence of severe disease between the each of the first three waves of virus circulation. Twenty-one clusters were reported during these three waves (4, 11 and 6 clusters, respectively), of which, 14 were considered to be possible human-to-human transmission.

**Conclusion:**

Though our case investigation for the first three waves found few differences between the epidemiologic and clinical characteristics, there is continued international concern about the pandemic potential of this virus. Since the virus continues to circulate, causes more severe disease, has the ability to mutate and become transmissible from human-to-human, and there is limited natural protection from infection in communities, it is critical that surveillance systems in China and elsewhere are alert to the influenza H7N9 virus.

## Background

Avian influenza A(H7N9) virus (referred to as H7N9 hereafter) was detected in mainland China in March 2013 with the identification of three severely ill patients with unexplained pneumonia [1–3]. This virus had not previously been detected in humans and posed a potential for pandemic spread [4]. At the time of its emergence, little was known about the virus including the spectrum and severity of illness, risk factors for infection and severity, transmissibility from person-to-person, and geographic distribution of H7N9 in humans and animals. To better understand this virus, active case monitoring and environmental surveillance were initiated.

Per Chinese notifiable disease reporting guidelines [5, 6], H7N9 positive cases by real-time reverse transcriptase polymerase chain reaction (RT-PCR), conventional RT-PCR, virus isolation, or a 4-fold rise in H7N9 antibody titers in serology are reported to the National Health and Family Planning Commission (NHFPC). After a case is identified, active surveillance to determine exposure history and contact monitoring is initiated by the local Center for Disease Control and Prevention (CDC). As part of this investigation, specimens from possible exposure locations (e.g., live poultry markets (LPM), commercial poultry farms, or bird feeding areas) are collected and tested for H7N9. Further, in some provinces routine environmental surveillance in LPMs is conducted by local CDCs to monitor viruses in the environment and to provide evidence for pandemic risk assessment [7, 8]. For this surveillance, specimens are collected from various locations and stalls in LPMs and tested for influenza viruses throughout the year.

Since H7N9 emerged, there have been three distinct waves of circulation emerging in the northern hemisphere typical winter months. In this study, we examined differences and similarities between these three circulation waves to inform future prevention and control measures.

## Methods

The first wave of H7N9 virus circulation is defined as detected cases with onset dates from March 31 to September 30, 2013. The second wave of H7N9 virus circulation is defined as detected cases with onset dates from October 1, 2013 to September 30, 2014. The third wave of H7N9 virus circulation is defined as detected cases with onset dates from October 1, 2014 to September 30, 2015. In this manuscript, we compare the first three waves of virus circulation in mainland China by examining the epidemiology, geographic distribution, clinical severity, the possible person-to-person spread, and seasonality of this virus.

For this study, suspected and confirmed cases were defined per World Health Organization (WHO) guidelines [1]. Most cases were identified through the Pneumonia of Unknown Etiology (PUE) surveillance system which identifies severe pneumonia cases without a known cause of illness [3]. In response to H7N9 emergence, the NHFPC issued the Protocol for the Prevention and Control of H7N9 which established enhanced surveillance for suspected H7N9 cases [5, 6], including oversampling of influenza-like illness (ILI) cases at sentinel hospitals participating in the National Influenza Surveillance System [9, 10], the implementation of a two week enhanced surveillance for all ILI case-patients in the counties or districts where H7N9 cases were detected, and monitoring of close contacts of confirmed H7N9 cases leading to the identification of additional cases. Further, cases were identified less commonly through routine multi-respiratory pathogen surveillance and screening for individuals showing signs of ILI at provincial and international borders.

A severe case was defined as having any of the following: a chest x-ray indicative of multi-lobar lesions or a >50% increase in the size of the lesions within a 48 h period; dyspnea or a respiratory rate of greater than 24 times per minute for adults; severe hypoxia defined as a less than or equal to 92% oxygen saturation while receiving 3–5 l of supplemental oxygen per minute; or shock, acute respiratory distress syndrome, or multiple organ dysfunction syndrome [11]. Non-severe cases were defined as those who did not meet the criteria for severity.

To examine the person-to-person transmissibility of this virus, we examined data from contact investigations and case clusters. Active field investigations were conducted for each H7N9 case per guidelines established by the China CDC. Case contacts were actively monitored for 7 days for respiratory symptoms after the last unprotected exposure to the case. Any contacts that developed fever (axillary temperature ≥37.5 °C) and any respiratory symptoms were isolated and specimens were collected and tested for H7N9. Any two cases with an epidemiologic link either with a common exposure or contact with an H7N9 case were considered part of a cluster [5, 6].

Data collection for H7N9 confirmed cases and their close contacts were determined by the NHFPC to be part of a continuing public health outbreak investigation and exempt from institutional review board assessment. Case data were obtained from the China Information System for Disease Control and Prevention (CISDCP), the notifiable diseases data system, and the H7N9 avian influenza information system, the web-based database for clinical and epidemiological information, and from epidemiological reports from field investigations of confirmed H7N9 cases. To compare the characteristics from the three waves, we used simple descriptive statistics and tests of proportions to determine statistical significance between the waves. Non-parametric tests, including the Wilcoxon and Kruskal Wallis tests were used to compare continuous variables such as age and time between illness onset and other dates of interest. Chi-square tests were used to compare frequencies of demographic and other categorical variables for the three waves. SPSS 17.0 (SPSS Inc., Chicago, IL, USA) was used for analyses.

## Results

### Epidemiologic characteristics comparison

**Fig. 1:**
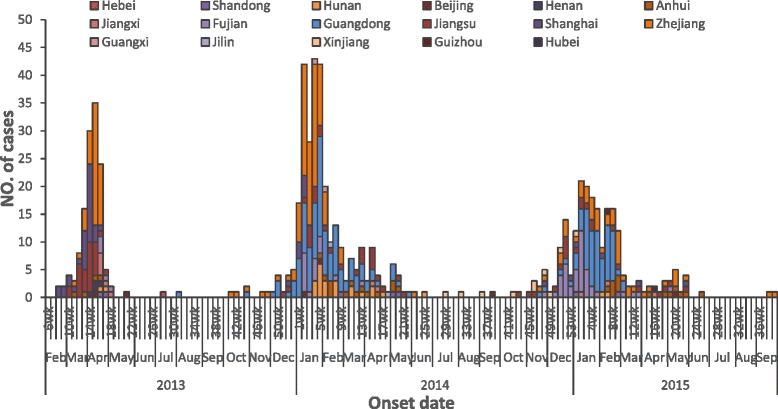
Epidemic Curve of Confirmed Influenza A(H7N9) Case-Patients in mainland China by Province and Date of Onset of Illness

**Fig. 2:**
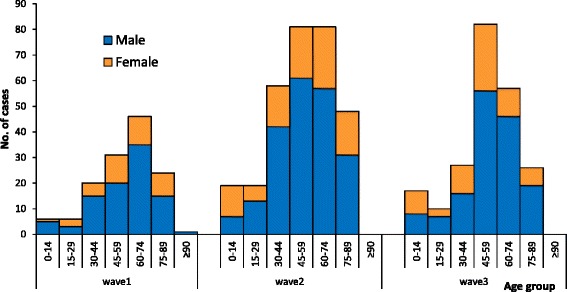
Gender Comparison of Case-Patients for Wave 1 and Wave 2 of Influenza A(H7N9) Virus Circulation

During the first wave, 134 cases were identified and reported to the NHFPC in mainland China. From emergence of the virus in February through May 2013, the virus rapidly spread through eastern mainland China impacting several provinces and municipalities (Fig. 1). Ninety-six percent of cases were reported in April and May 2013. After the initial peak activity in April and May 2013, there was decline in circulation with only two cases and one death reported between June and September 2013. The majority of case-patients were older men with underlying health conditions and most experienced severe diseases. During the first wave, 70% of cases were men (*n* = 94) (Fig. 2) and the median age was 61 years (range: 3–91 years). Forty-four died resulting in a case fatality proportion (CFP) of 33% (Table 1).

**Table 1:** Gender and Age Comparison of Influenza A(H7N9) Case-Patients and Deaths in Mainland China During the First (March 31-September 30, 2013), Second (October 1, 2013 – September 30, 2014) and Third (October 1, 2014 – September 30, 2015) Waves of Circulation

Age (Years)	Total		Wave 1		Wave 2		Wave 3	
	N (%)	Death (CFP%)	N (%)	Death (CFP%)	N (%)	Death (CFP%)	N (%)	Death(CFR%)
0–14	42(6.4)	1(2.4)	6(4.5)	0(0)	19(6.2)	0(0)	17 (7.8)	1 (5.9)
15–29	35(5.3)	11(31.4)	6(4.5)	1(16.7)	19(6.2)	7(36.8)	10 (4.6)	3 (30.0)
30–44	106(16.1)	26(24.5)	20(14.9)	3(15.0)	58(19.0)	14(24.1)	28 (12.8)	9 (32.1)
45–59	193(29.3)	75(38.9)	31(23.1)	7(22.6)	81(26.5)	31(38.3)	81 (37.0)	37 (45.7)
60–74	182(27.6)	96(52.7)	46(34.3)	19(41.3)	81(26.5)	47(58.0)	55 (25.1)	30 (54.5)
75–89	100(15.2)	60(60)	24(17.9)	13(54.2)	48(15.7)	29(60.4)	28 (12.8)	18 (64.3)
≥90	1(0.2)	1(100)	1(0.8)	1(100)	0(0)	-	0 (0)	
Total	659	270 (41.0)	134	44(32.8)	306	128(41.8)	219	98 (44.7)
Age median (range)	57 (9 months-91)	62 (13–91)	61 (2–91)	66.5 (27–91)	56 (1–88)	63 (20–86)	56 (9 months-88)	59 (13–83)

*CFP* Case Fatality Proportion

During the second wave, there were 306 cases and 128 deaths (CFP: 42%) in mainland China (Table 1). The peak in reported cases occurred between January and February 2014 (Fig. 1). The number of reported cases more than doubled from the first wave to the second wave. Men accounted for 69% (*n* = 211) of reported cases (Fig. 2) and this proportion was similar to the proportion of male case-patients in the first wave (70% vs. 69%, *p* = 0.80). The median age of case-patients during the second wave was 58 years (range: 1–88 years) which was slightly lower compared to the first wave (*p* = 0.03).

During the third wave, there were 219 cases and 98 deaths (CFP: 45%) in mainland China (Table 1). The peak in reported cases occurred between January and February 2015 (Fig. 1). The number of cases was less compared to the second wave. Men accounted for 69% (*n* = 152) of reported cases during the third wave (Fig. 2) which was similar to the previous two waves (69% vs. 70%, 69%, *p* = 0.97). The median age of case-patients reported in the third wave was 56 years (range: 9 months-88 years) which was slightly lower compared to the first wave (*p* = 0.01), and was similar to the second wave (*p* = 0.69). The CFP of cases increased from 33% (first wave) to 42% (second wave) and 45% (third wave), there was no statistically significant difference of CFPs between the three waves (*p* = 0.08). The proportion of deaths was highest among cases 60 years or older for the second and third waves compared to the first wave (46% vs. 59%, 57%). During the first wave, the highest CFPs were among cases 60–74 years of age (41%) and 75–89 years of age (54%). In the second wave, the CFPs for the same age groups were 58% and 60% and in the third wave, the CFPs were 55% and 64% for the same age groups. No deaths among children less than 15 years of age were reported in the first two waves and 1 death in a case-patient less than 15 years of age was reported in the third wave. Among cases who died, the median age was 66.5, 63.0 and 58.5 years in each of the three waves, respectively. The median age of death in the third wave was lower than in the first wave (*p* = 0.001) and was similar to the second wave (*p* = 0.06). The CFP for males and females was similar between these three waves (*p* = 0.28 and *p* = 0.25, respectively).

### Exposure comparison

During the first three waves, the majority of cases reported exposure to poultry or LPMs. Exposure to poultry was defined as direct contact with live poultry such as feeding, cleaning, slaughtering or exposure to the environment where poultry were kept (e.g., backyard or visiting a live poultry market). Of 127 (95% of cases) first wave cases with exposure history information, 26 (21%) reported direct poultry exposure and 84 (66%) reported visiting of LPMs prior to illness onset (Table 2). Among those cases visiting LPMs, 15 (18%) reported working at a LPM and 69 (82%) reported only visiting the LPM. Thirteen cases in the first wave (10%) reported no exposure to poultry or LPMs. Four (3%) reported exposure to other H7N9 case-patients with no other known exposure implying possible limited human-to-human transmission. Among 296 (97%) second wave cases with exposure history information, 56 (19%) reported exposure to poultry and 203 (69%) reported visiting a LPM prior to illness onset. Among those patients visiting LPMs, 40 (20%) reported working at a LPM, 163 (80%) reported only visiting a LPM. Seven (2%) reported exposure to other H7N9 case-patients with no other known exposure, implying possible human–to-human transmission and 3 (1%) reported both exposure to other H7N9 case-patients and common poultry exposure with reported H7N9 case-patients. Twenty-seven (9%) reported no poultry or LPM exposure. Among 207 (95%) third wave cases with exposure history information, 50 (24%) reported exposure to poultry and 136 (66%) reported visiting a LPM prior to illness onset. Among those patients visiting LPMs, 12 (9%) reported working at a LPM, 124 (91%) reported only visiting a LPM. Four (2%) reported exposure to other H7N9 case-patients with no other known exposure implying possible human-to-human transmission and 1 (1%) reported both exposure to other H7N9 case-patients and common poultry exposure. Sixteen (8%) reported no poultry or LPM exposure. There was no statistically significant differences in poultry or LPM exposure of cases among between these three waves (87%, 88%, 90% *p* = 0.614).

**Table 2:** Exposure History of Influenza A(H7N9) Case-Patients During the First (March 31-September 30, 2013), Second (October 1, 2013 – September 30, 2014) and Third (October 1, 2014 – September 30, 2015) Waves of H7N9 Virus Circulation

Exposure	Wave 1(*N* = 127)	Wave 2(*N* = 296)	Wave 3(*N* = 207)
Exposure to poultry	26 (20.5)	56 (19.0)	50 (24.2)
Exposure to live bird markets (LPM)	84 (66.1)	203 (68.6)	136 (65.7)
Working at a LPM	15 (17.9)	40 (19.7)	12 (8.8)
Visiting of LPMs	69 (82.1)	163 (80.3)	124 (91.2)
No exposure to poultry	13 (10.2)	27 (9.1)	16 (7.7)
Possible limited H-H transmission	4 (3.1)	7 (2.3)	4 (1.9)
Both possible limited H-H transmission and common exposure to poultry	-	3 (1.0)	1 (0.5)

*H-H* Human-to-Human Transmission

### Geographic comparison

During the first three waves, 661 cases were reported by 543 townships, spanning 282 counties or districts, 92 cities and 17 provinces in mainland China. During the first wave, cases were reported by 112 townships, spanning 71 counties or districts, 29 cities and 12 provinces. During the second wave, cases were reported by 266 townships, from 165 counties or districts, 65 cities and 14 provinces. Ten provinces, 18 cities, 26 counties or districts and 9 townships reported cases in both waves. During the second wave, 4 provinces (Guangxi, Guizhou, Jilin, and Xinjiang) reported cases for the first time. Additionally, there were 47 cities, 139 counties or districts, and 257 townships that reported cases for the first time during the second wave. During the third wave, 219 cases were reported by 201 townships, spanning 138 counties or districts, 64 cities and 13 provinces in mainland China. During the third wave, 1 province (Hubei) reported cases for the first time. Additionally, there were 16 cities, 72 counties or districts, and 174 townships that reported cases for the first time during the third wave.

**Fig. 3:**
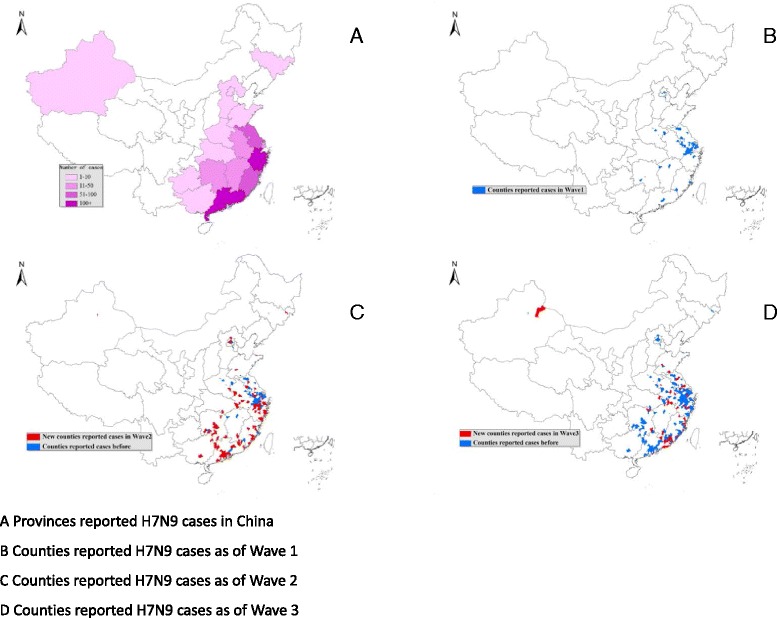
Geographic Distribution of Avian Influenza A(H7N9) Cases. **a** Provinces reported H7N9 cases in China. **b** Counties reported H7N9 cases as of Wave 1. **c** Counties reported H7N9 cases as of Wave 2. **d** Counties reported H7N9 cases as of Wave 3

The first wave cases were concentrated in Zhejiang and Jiangsu Provinces and Shanghai municipality along the Southeast Coast of mainland China accounting for 80% of total reported cases (Fig. 3). The geographic distribution shifted further south during the second wave with Guangdong and Zhejiang Provinces accounting for 66% of total cases reported (Table 3). During the third wave, Guangdong and Zhejiang were still the two provinces that reported the most cases, accounting for 54% of total number. And Fujian Provinces reported 19% cases of the total number. The H7N9 outbreaks during these three waves are considered to be geographically sporadic since cases have been reported by 543 different townships, the lowest administrative level. Of those townships, 462 reported only one case, 62 reported two cases, 10 reported three cases, and 9 reported more than four cases. Beyond this main geographic distribution of cases, two other provinces beyond the main impacted reported cases in the second and third waves. Jilin province in North-East China reported 2 cases in the second wave and Xinjiang in North-West China reported 10 cases in both the second and third waves.

**Table 3:** Human Influenza A(H7N9) Confirmed Case-Patients in Mainland China During the First (March 31-September 30, 2013) , Second (October 1, 2013 – September 30, 2014) and Third (October 1, 2014 – September 30, 2015) Waves of the H7N9 Virus Circulation, by Province

Provinces	Total		Wave 1		Wave 2		Wave 3	
	Cases	Deaths	Cases	Deaths	Cases	Deaths	Cases	Deaths
Zhejiang	185	71	45	10	93	38	47	23
Guangdong	182	68	1	0	109	37	72	31
Jiangsu	78	36	29	10	27	13	22	13
Fujian	63	17	5	0	17	5	41	12
Shanghai	47	28	33	18	8	7	6	3
Anhui	32	18	4	2	14	11	14	5
Hunan	26	11	2	1	22	9	2	1
Jiangxi	11	1	6	1	2	0	3	0
Xinjiang	10	9	0	0	3	2	7	7
Shandong	7	3	2	0	3	2	2	1
Beijing	6	2	2	0	3	1	1	1
Henan	4	1	4	1	0	0	0	0
Guangxi	2	1	0	0	2	1	0	0
Jilin	2	1	0	0	2	1	0	0
Guizhou	2	2	0	0	1	1	1	1
Hebei	1	1	1	1	0	0	0	0
Hubei	1	0	0	0	0	0	1	0
Total	659	270	134	44	306	128	219	98

### Severity comparison

Of 128 first wave cases with severity data, 110 (86%) were considered severe, 264 (87%) of 303 cases were defined as severe during the second wave, and 175 (86%) of 204 cases in the third wave were considered severe. There was no statistically significant difference among the severity of cases during these waves (*p* = 0.894). Although the proportion of non-severe cases did not vary between the waves, during the second wave there was an observed increase in the number of non-severe cases identified. Forty-nine percent of non-severe cases identified during the second wave occurred in Guangdong province (*n* = 19). And in the third wave, 6 provinces reported 29 non-severe cases, of which Guangdong reported 15 (52%) cases and Fujian reported 6 (21%) cases. During all waves combined, non-severe disease was more common among children <15 years of age (41/42, 98%) than adults > =15 years (32/406, 7%). However, the percent of children with non-severe disease was similar during the three waves (6/18, 33%; 19/39, 49%; 16/29 55%; *p* = 0.341).

Nearly all of the case-patients from the waves reported fever (97, 94 and 94%, respectively; Table 4). The next most common symptom reported was cough (71, 86 and 82%, respectively). Of 466 case-patients from all three waves with information about underlying health conditions, 52% had at least one underlying condition. The most commonly reported conditions were cardiovascular or cerebrovascular disease (34%), metabolic diseases (15%) and chronic lung disease (11%). There was no statistically significant difference in the percent of cases with underlying health conditions among the three waves: 58%, 52% and 49% (*p* = 0.408).

**Table 4:** Clinical Characteristics of Avian Influenza A(H7N9) Case-Patients by Virus Circulation Wave

	Total (N,%)	Wave 1 (n, %)	Wave 2 (n, %)	Wave 3 (n, %)	*P*
Main symptoms at early stage (N1 = 76,N2 = 227,N3 = 169)					
Fever	445(94.3)	74(97.4)	213(93.8)	158(93.5)	0.444
Cough	386(81.8)	54(71.1)	194(85.5)	138(81.7)	0.019
Weakness	186(39.4)	29(38.2)	88(38.8)	69(40.8)	0.891
Muscle soreness	107(22.7)	20(26.3)	53(23.4)	34(20.1)	0.532
Others^a^	170(36)	37(48.7)	70(30.8)	63(37.3)	0.018
Underlying medical conditions (N1 = 74,N2 = 226,N3 = 166)	242(51.9)	43(58.1)	118(52.2)	81(48.8)	0.408
Cardiovascular and cerebrovascular disease	159(34.1)	29(39.2)	69(30.5)	61(36.8)	0.266
Metabolic diseases	68(14.6)	12(16.2)	37(16.4)	19(11.5)	0.359
Chronic lung disease	50(10.7)	12(16.2)	22(9.7)	16(9.6)	0.251
Chronic liver diseases	32(6.9)	3(4.1)	20(8.9)	9(5.4)	0.241
Hematological system diseases	14(3.0)	1(1.4)	9(4)	4(2.4)	0.441
Cancer	10(2.1)	2(2.7)	6(2.7)	2(1.2)	0.580
Rheumatic autoimmune disease	11(2.4)	5(6.8)	2(0.9)	4(2.4)	0.015
Immunosuppressive state	12(2.6)	1(1.4)	5(2.2)	6(3.6)	0.529
Chronic kidney diseases	16(3.4)	1(1.4)	6(2.7)	9(5.4)	0.186
Nervous system or neuromuscular dysfunction	6(1.3)	0(0)	4(1.8)	2(1.2)	0.500
Complications (N1 = 71,N2 = 219,N3 = 162)	406(89.8)	64(90.1)	197(90)	145(89.5)	0.985
Pneumonia	392(86.7)	64(90.1)	187(85.4)	141(87.0)	0.585
Respiratory failure	311(68.8)	47(66.2)	144(65.8)	120(74.1)	0.195
Acute Respiratory Distress Syndrome(ARDS)	298(65.9)	42(59.2)	141(64.4)	115(71)	0.171
Hepatic insufficiency	193(42.7)	25(35.2)	90(41.1)	78(48.2)	0.148
Renal insufficiency	152(33.6)	23(32.4)	68(31.1)	61(37.7)	0.391
Septic shock	141(31.2)	14(19.7)	64(29.2)	63(38.9)	0.010
Cardiac failure	124(27.4)	16(22.5)	49(22.4)	59(36.4)	0.006
Neurologic complications	27(6.0)	2(2.8)	12(5.5)	13(8.0)	0.277
Disseminated intravascular coagulation	25(5.5)	3(4.2)	9(4.1)	13(8.0)	0.222
Rhabdomyalysis	17(3.8)	1(1.4)	6(2.7)	10(6.2)	0.115
Failure of two or more organs	302(66.8)	45(63.4)	139(63.5)	118(72.8)	0.127

N1: Number of individuals in Wave 1 who had clinical information, N2: Number of individuals in Wave 2 who had clinical information, N3: Number of individuals in Wave 3 who had clinical information

^a^Including shortness of breath, chest distress, expectoration, nausea, vomiting, et al

Among case-patients, 90% were reported to have at least one complication due to H7N9 virus infection. The most common complications from infection included pneumonia (87%), respiratory failure (69%), ARDS (66%), and hepatic insufficiency (43%). More than half of cases (302, 67%) developed multiple organ failure. There were no statistically significant differences in any of the individual complications experienced between the two waves except Septic shock and Cardiac failure (Table 4).

We also compared the time durations from illness onset to different important epidemiological time points in the course of clinical illness such as: first seeking healthcare, hospitalization, lab confirmation, initiation antiviral treatment, and death between the three waves. The time between illness onset and first seeking healthcare was 1 day (IQR:0–3 days) for the first wave, 1 day (IQR: 0–4 days) for the second wave, and 1 day (IQR: 0–3 days) for the third wave. The time between illness onset and hospital admission was 5 (IQR: 4–7) days, 5 (IQR: 3–7) days, and 5 (IQR: 3–7) days for three waves, respectively. The time between illness onset and lab confirmation was 8 (IQR: 6–11), 8 (IQR: 6–11) and 8 (IQR: 6–10) days for three waves, respectively. The median time between illness onset to initiation of antiviral treatment and to death changed from 7 days (antivirals) and 21 days (death) in the first wave to 6 days and 19 days and 6 days and 16 days in the second and third waves, respectively (Table 5). There were no statistically significant differences for the time from illness onset to hospitalization, case confirmation, initiation of antiviral treatment, and death between three waves of circulation.

**Table 5:** Comparison of time duration from illness onset to different points among different waves

Time duration (days)	wave 1		wave 2		wave 3		*P*
	No. of cases	Median (IQR)	No. of cases	Median (IQR)	No. of cases	Median (IQR)	
Time between illness onset to first seeking healthcare	127	1 (0–3)	255	1 (0–4)	167	1 (0–3)	0.37
Time between illness onset to hospitalization	75	5 (4–7)	239	5 (3–7)	185	5 (3–7)	0.22
Time between illness onset to lab confirmation	133	8 (6–11)	306	8 6–11)	219	8 (6–10)	0.07
Time between onset to initiation antiviral treatment	50	7 (5–9)	195	6 (4–9)	151	6 (4–8)	0.17
Time between illness onset to death	44	21 (11–34)	127	19 (10–31)	98	16 (10–27)	0.23

### Evidence of person-to-person transmission

During the three waves of virus circulation, case clusters were identified and an investigation was conducted to determine either common exposure or if possible human-to-human transmission between the cases. Twenty-one clusters of confirmed cases were reported during these three waves (4, 11 and 6 clusters). Twenty of the clusters each included 2 cases and one cluster included 3 cases. These 21 clusters were thought to be possible human-to-human transmission or to have a common epidemiologic exposure. There was no statistically significant difference in the proportion of case-patients that occurred in clusters during the waves (6, 8 and 5% of reported case-patients, *p* = 0.62). Fourteen clusters were considered to be possible human-to-human transmission (which meant there were 14 cluster index cases and 15 possible secondary cases), 3 clusters were likely a common epidemiologic exposure, and for 4 clusters the investigation could not rule out a common exposure or confirm possible human-to-human transmission. For the 21 index cases of the clusters, 71% (15/21) were male and the median age was 49 years (range: 9 months-87 years). And for the 22 subsequently reported cases, 55% (12/22) were male, and the median age was 26.5 (range: 1–77 years). No statistical differences were observed for sex and age between index and subsequently-reported cases (*p* = 0.25, *p* = 0.14). All 21 index cases (including 14 possible human-to-human transmission cluster index cases) had live poultry related exposure history before illness onset, including 17 cases who had live poultry market exposure or were exposed to poultry brought from a market, and 4 cases were exposed to backyard poultry. Two clusters (each including 2 cases) of 21 clusters occurred in the hospital settings as cases were admitted to the same ward before the index case in the cluster was diagnosed with avian influenza A(H7N9) virus infection. The remaining 19 clusters were considered family clusters with 2 cases in each of 18 clusters and 3 cases in one cluster. Among cases in these clusters, 75% (15/20) of subsequently-reported cases had a blood relationship with the respective cluster index case. Given the limited number of clusters with possible evidence of human-to-human transmission and the more frequent occurrence of geographically sporadic occurrence of cases, we conclude that there is no evidence of sustained human-to-human transmission of this virus.

### Seasonality

For wave 1, illness onset began in February and extended through May of 2013, beyond the typical colder or winter months (Fig. 1). During the second wave, cases occurred significantly earlier (median date onset 7 April 2013 for wave 1 vs 26 January 2014 for wave 2), beginning in December 2013 and declining by February 2014. During the third wave, continued reporting cases occurred in November 2014, earlier than the second wave, and declined by February 2015. These findings imply that the first wave occurred at the time of virus emergence, but that the second and third wave cases occurred during the colder or winter months, which is typical seasonality for avian influenza viruses.

## Discussion

Our evaluation revealed more similarities than differences between the first three waves of virus circulation. The proportion of males, the age and gender distribution, the clinical severity and characteristics were similar. Human cases also continued to be sporadic with only a few cases occurring in each of the communities. Differences included a slightly higher median age in the first wave compared with the other two waves. There was a slight shift in the geographic distribution of cases from the southeast coast to more southern mainland China. Further, the timing of onset dates differed between the waves shifting initially from February-April to December-March. In the epidemic curve of H5N1 showed in the WHO monthly report [12], we figured that H5N1 avian influenza epidemics are more likely to circulate during colder or winter months. However, the H7N9 virus first emerged in late Winter/early Spring 2013 before shifting to the typical observed seasonality for avian influenza viruses. In the first wave, the virus emerged in the February and peaked in April 2013, whereas the second wave began earlier in the winter season with increasing number of cases in December 2013, a peak in January 2014 and a rapid decline in cases by March 2014. Additional waves of avian influenza A(H7N9) are needed to support our assumption that this virus tends to circulate in colder, winter months similar to other avian influenza viruses, such as H5N1.

There are several possible reasons for these similarities between the waves. First, the H7N9 viruses circulating during the first three waves were antigenically similar with a few point mutations causing no change in the pathogenicity in the internal genes. No substitutions in key critical binding positions and no antigenic changes that impact infectiousness or resistance to neuraminidase inhibitors were identified (China CDC, unpublished data). Second, there were no observed changes in exposure risk prior to illness onset. The main exposures included visiting LPMs or poultry contact and were reported by >80% of cases in all waves [1, 2]. Additionally, the demographic characteristics of cases who generally visit LPMs did not change between the three waves.

Among the differences between the three waves was a shift in the geographic distribution of cases. During the first wave, many of the impacted administrative areas instituted certain control measures to reduce transmission including policies to improve conditions at LPMs, such as temporary closure, routine scheduled standard cleaning and disinfection practices, and culling of infected flocks [13–17]. These measures may have resulted in fewer cases in those provinces during the second wave. For example, Shanghai closed the LPMs from the beginning of the Spring Festival, January 31 to April 30, 2014 [18]. At the end of this period, only a limited number of Shanghai markets were re-opened and these were required to follow strict rules regarding periodic closure and routine cleaning and disinfection as well as limits to the movement of poultry. The number of cases reported by Shanghai Municipality was much lower during the second wave than the first wave (China CDC, unpublished data).

The shift in geographic distribution may also have been due to natural spread, especially among poultry, to areas not affected in the first wave, especially southern China. Further, this virus spread beyond LPMs to farm flocks in one northern province that borders North Korea and Russia [16]. During the second wave, Jilin Province reported its first human case. The identification of the case occurred first prompting an environmental investigation with specimen collection at both poultry farms and nearby suppliers. From this investigation, H7N9 positive poultry and environmental specimens were identified in a farm in a north province of the previously not impacted areas [19]. In addition, H7N9 cases were identified in Xinjiang Province, along the western border of China during the second wave and Hubei in central China during the third wave. These findings underscore the importance of surveillance and monitoring for the spread of this virus as well as the difficulty of identifying this virus in poultry flocks before the occurrence of human cases.

The number of cases was much higher in the second wave, including both more severe and non-severe cases compared with both the first and third wave. This increase may have been due to early and increased recognition of cases, and further spread of the virus to other parts of China. Non-severe cases may have been more likely to be identified in the second wave because of the two week enhanced surveillance for all ILI case-patients in the counties or districts where H7N9 cases were detected [6, 20]. Some counties or districts detected a higher number of non-severe cases through this mechanism (China CDC, unpublished data). Another possible reason for the detection of more non-severe cases may be the strict monitoring of close contacts. If a close contact developed respiratory symptoms, the contact was tested for H7N9. These tests might have occurred early and if the contact received timely treatment this may have led to non-severe illness [21]. In addition, some provinces established their own surveillance programs for respiratory pathogens. Thus, the enhanced systems in these provinces may be another reason for the increase in the number of detected non-severe cases during the second wave.

Although our findings suggested that the epidemic of H7N9 remains to be sporadic during the three waves in China, but the emergence of clusters emphasized the importance of continued surveillance for the cases and the contact tracing and management for close contacts of confirmed cases. Given that some clusters occurred in hospital settings, it highlights the importance of possible hospital acquired infections and the need for infection control measures for both prevention and control of possible outbreaks of this virus and other viruses within hospital wards.

For context to the surveillance program in China, the National ILI Surveillance System requires participating sentinel hospitals in southern provinces to collect specimens from 5 to 15 ILI case-patients per week throughout the year. In Northern provinces, 10–15 specimens per week during the typical influenza season (October to March) and 5–15 per month from April to September. However, due to the H7N9 emergence, all provinces were required to increase testing 20 specimens in each surveillance time unit as a requirement of the May 10, 2013 [6]. The increased surveillance activities may have contributed to the increased detection of H7N9 human cases.

There are some limitations of our study. Our comparison relies on data from cases that have been both detected and reported. There were likely some cases that were not detected and other cases may have been detected but not reported. The main system that detects respiratory emerging infectious diseases is the PUE system [3] which may under estimate the true number of cases since only severe illness is reported. This may have resulted in a bias towards greater detection and reporting of severe compared with non-severe cases. Further, because detection required a medically attended illness, it is possible some cases were missed if the individual did not seek medical care. However, given the high awareness of this virus, it is likely that very few patients with severe illness were missed. Our comparison is likely an accurate reflection of cases with severe illness, but may not fully depict non-severe illness.

To address these limitations, community studies are needed to determine if non-severe illness is more common than detected and to further determine if the virus has spread beyond the geographic area where human cases have been reported. One serologic study conducted by China CDC from May to June 2013 found no seropositive cases of H7N9 among serum specimens collected from individuals living in a rural area of Jiangxi Province where at least one human H7N9 case was identified (China CDC, unpublished data). This finding suggests that infection was uncommon among individuals living in the same village as the case-patient and the virus may not be present in the community (or backyard flocks) but rather limited to areas where there is exposure to LPMs or commercial poultry. In contrast, other serological studies found that some individuals with occupational exposure to poultry were seropositive for the virus, but had no recent clinical signs or symptoms of H7N9 virus infection [22, 23]. In one such serologic study conducted in April and May 2013 in Zhejiang Province, 6.3% (25/396) of sampled poultry workers had hemagglutination-inhibition (HI) antibody titers of ≥80 indicating prior H7N9 infection [22]. In a separate study among poultry workers conducted in May and December of 2013 in Shenzhen, Guangdong Province, 36/501 (7.2%) and 56/375 (14.9%) were found to have an HI antibody titer ≥1:160 to H7N9, indicating a substantial increase in the seroprevalence of antibodies against H7N9 between the two different sampling periods [20]. Further, of 96 individuals who participated in both surveys, 52 (54.2%) poultry workers had a seroconversion ≥4‐fold rise in H7N9 antibody titers from May to December implying serologic evidence of infection during the six months between surveys [20]. This finding may be explained by the duration and type of poultry exposure as well as exposure to other avian influenza viruses. Findings from these serologic studies imply that the risk of H7N9 may not be high in the community, but individuals with extended exposure, such as poultry workers, may be at high risk for infection, which is most often asymptomatic.

From our study we found that most H7N9 cases had LPMs exposure history, and in Zhejiang Province and Guangdong Province, routine environmental surveillance showed that LPMs were the most contaminated place by H7N9 virus, and the detection of H7N9 virus spiked in cold months [7, 8], which was in accordance with the epidemic trends of the H7N9 in mainland China. And several other subtypes of human infections of avian influenza have also been detected in China, including H5N1, H5N6, H10N8, and H9N2 et al. Most of the infections also resulted from the live poultry exposure (sick/dead/healthy-looked poultry) or poultry related environment exposure, especially live poultry markets [24] (China CDC, unpublished data), which indicated that LPMs in China played an important role in the human infections of avian influenza virus. However, before such a drastic measure can be implemented, first prevention and control measures including the strict cleaning and disinfection measures should be taken in the markets to reduce the infection risk of residents, which is suggested in the national technical guideline [20]. Additionally, poultry workers should wear personal protective equipment when dealing with poultry to protect their health and also to limit the possible spread of virus. These measures should be evaluated to determine if they are effective in reducing transmission or if more drastic prevention methods are necessary to protect the health of the population. Further, we suggest that people avoid direct contact with live poultry, especially poultry in the markets. In addition to these control measures, studies should be implemented such as serological studies to understand the full spectrum of illness, genetic studies to better understand changes in the virus, perhaps household transmission studies to better understand human-to-human transmission, et al.

There is currently no evidence that this virus can spread efficiently from person-to-person. Data from reported cases show that H7N9 virus infection commonly causes very severe illness and death. Although there has been vigilant monitoring of this virus to identify changes in disease characteristics, no significant changes have been observed. Should the virus change to become more transmissible from person-to-person, the next global influenza pandemic could result, leading to significant morbidity and mortality. Public health agencies are continuing to monitor the epidemiology of this virus to assist in planning control and prevention measures.

## Conclusions

Though our case investigation for the first three waves found few differences between the epidemiologic and clinical characteristics, there is continued international concern about the pandemic potential of this virus. Since the virus continues to circulate among poultry, causes more severe disease in humans, has the ability to mutate and become human-to-human transmissible, and there is limited natural protection from infection in human populations, it is critical that surveillance systems in China and elsewhere are monitoring for continued geographic spread or introduction of this virus in poultry or persons in their populations.

## Abbreviations

CDC: Center for Disease Control and Prevention
CISDCP: China Information System for Disease Control and Prevention
HI: Hemagglutination-inhibition
ILI: Influenza-like illness
LPM: Live poultry markets
NHFPC: National Health and Family Planning Commission
PUE: Pneumonia of Unknown Etiology
RT-PCR: Real-time reverse transcriptase polymerase chain reaction
WHO: World Health Organization
